# Spatial transcriptomic analysis reveals spatial features in human Stanford type A aortic dissection

**DOI:** 10.1016/j.isci.2026.116868

**Published:** 2026-07-16

**Authors:** Yan-Hong Li, Ying Cao, Fen Liu, Li-Ying Wang, Hai-zhou Liu, Qian Zhao, Dilare Adi, Qiang Huo, Zheng Liu, Jun-Yi Luo, Xiao-Mei Li, Di Liu, Yi-Ning Yang

**Affiliations:** 1 Clinical Laboratory, The First Affiliated Hospital of Xinjiang Medical University, Urumqi 830054, China; 2 Computational Virology Group, Center for Bacteria and Virus Resources and Application, Wuhan Institute of Virology, Chinese Academy of Sciences, Wuhan 430071, China; 3 Xinjiang Key Laboratory of Cardiovascular Disease, Clinical Medical Research Institute, The First Affiliated Hospital of Xinjiang Medical University, Urumqi, Xinjiang 830054, China; 4 Department of Cardiology, The First Affiliated Hospital of Xinjiang Medical University, Urumqi 830054, China; 5 University of Chinese Academy of Sciences, Beijing 101408, China; 6 Department of Cardiac Surgery, The First Affiliated Hospital of Xinjiang Medical University, Urumqi 830054, China; 7 Department of Cardiology, People’s Hospital of Xinjiang Uyghur Autonomous Region, Urumqi 830001, China; 8 Xinjiang Key Laboratory of Cardiovascular Homeostasis and Regeneration Research, Urumqi 830001, China

**Keywords:** Stanford type A aortic dissection, spatial transcriptomics, *SPP1* signaling

## Abstract

Stanford type A aortic dissection (AAD) is a life-threatening cardiovascular disease characterized by tearing in the aortic wall. Using spatial transcriptomics and multiplex immunofluorescence, we comprehensively analyzed ascending aortas from eight AAD patients across different severities and segments. We demonstrate that SPP1-driven inflammatory signaling intensifies with AAD severity, identifying a nine-gene, layer-anchored severity scale: MYL6/CALD1/MYH9 (mild); CCL2/CP/COL4A1 (moderate); and TMSB4X/ATP5F1E/PKM (severe). Importantly, the collagen-remodeling triad COL1A1/COL3A1/MMP2 is concurrently up-regulated in the brachiocephalic, left subclavian, and left common carotid arteries, often before the ascending aorta meets surgical diameter thresholds. These molecular signatures provide a critical foundation for non-invasive biomarker discovery, risk stratification, and precision pharmacotherapy targeting the SPP1-inflammatory axis, ultimately offering new insights into AAD mechanisms and therapeutic targets.

## Highlights

• Spatial transcriptomics maps cellular dynamics in human aortic dissection
• *SPP1*-driven inflammatory signaling intensifies with progressive disease severity
• A nine-gene, layer-anchored signature stratifies AAD into three severity stages
• *COL1A1*, *COL3A1*, *and MMP2* upregulation in branch vessels precedes surgical limits

## Graphical abstract

**Figure:**
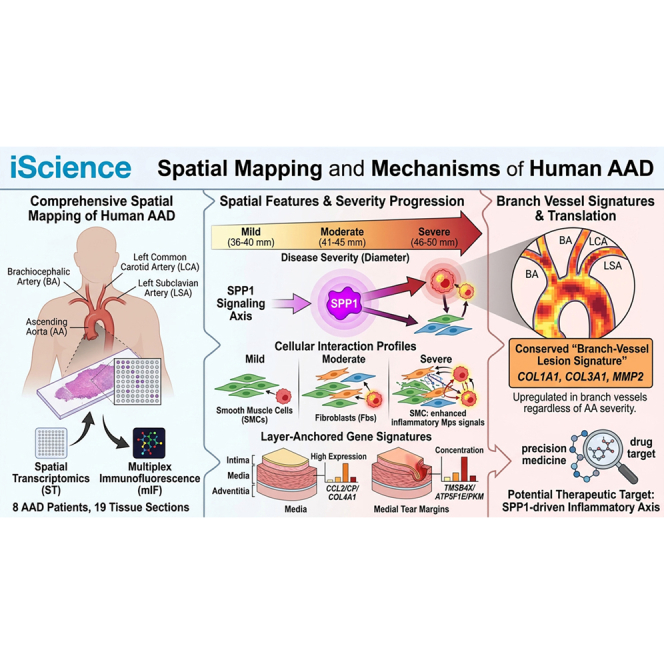

## Teaser

cardiovascular medicine; genomics; transcriptomics

## Introduction

Stanford type A aortic dissection (AAD) is a life-threatening cardiovascular disease with a high mortality rate without immediate surgical intervention.^1^ During the last decade, contemporary studies have reported an in-hospital mortality rate of 22%.^2^^,^^3^ While ascending-rupture dominates early mortality, up to 40% of patients present with primary tears in the arch or supra-aortic branches, and many harbor radiologically silent mural changes that only declare themselves months later. The weakening of the media layer is considered a primary cause of AAD, and there are multiple factors, including degradation of the extracellular matrix (ECM), hypertension, atherosclerosis, cellular phenotypic switching, and apoptosis^4^ that can lead to its rupture. However, due to technical limitations, the exact mechanisms underlying AAD pathogenesis remain unclear. Current animal models—whether utilizing surgical tearing, chemical induction, or gene editing—often fail to faithfully recapitulate the chronic, progressive pathology of human AAD. Instead, these models provide complementary insights into specific mechanistic aspects, highlighting the critical need to directly investigate complex pathological changes within human tissues.

The development of AAD involves complex, multilayered tissue architecture with abundant elastic fibers and unique cellular phenotypes and plasticity. Historically, conventional bulk RNA sequencing obscured crucial spatial and morphological contexts, making it difficult to accurately pinpoint localized molecular changes in AAD.^5^^,^^6^^,^^7^^,^^8^ In recent years, the significant emergence of sequencing-based spatial transcriptomics (ST) technologies has revolutionized this field. These innovative techniques provide a powerful tool for directly measuring genome-wide gene expression within tissue sections, enabling detailed interrogation of tissue biology at an unprecedented level.^9^^,^^10^ Vascular heterogeneity has emerged as a major focus in mechanistic studies of aortic disease physiology and pathology.^11^^,^^12^^,^^13^ Understanding the biological functions, networks, and interactions of different cell types that regulate the aorta and disease development requires both cellular information and spatial context.^14^ While various approaches have mapped cellular diversity in the mouse aorta—revealing distinct cell atlases across different compartments^15^^,^^16^^,^^17^—little is known about the spatial cellular complexity, relative contributions, and layer-specific molecular changes in the human AAD aorta.

Here, we conducted ST and multiplex immunofluorescence staining (mIF) to comprehensively analyze the ascending aortas from eight AAD patients with different severities and segments. We demonstrate that inferred *SPP1*-driven inflammatory signaling intensifies in lock-step with ascending-aortic dissection severity, and we derive a nine-gene, layer-anchored severity scale: *MYL6/CALD1/MYH9* for mild lesions; *CCL2/CP/COL4A1* for moderate disease; and *TMSB4X/ATP5F1E/PKM* for advanced wall failure. Importantly, the collagen-remodelling triad *COL1A1/COL3A1/MMP2* is concurrently up-regulated in the brachiocephalic, left subclavian, and left common carotid arteries, often before the ascending aorta meets surgical diameter thresholds. Rather than serving as immediate diagnostic tools, these spatially contextualized molecular signatures and inferred communication networks offer a crucial structural framework for understanding advanced aortic degeneration. They provide valuable candidate targets for future mechanistic investigations and translational research aimed at understanding the SPP1-inflammatory axis in AAD progression.

## Results

### Spatial transcriptomic analysis of the human aortic wall

**Figure 1:**
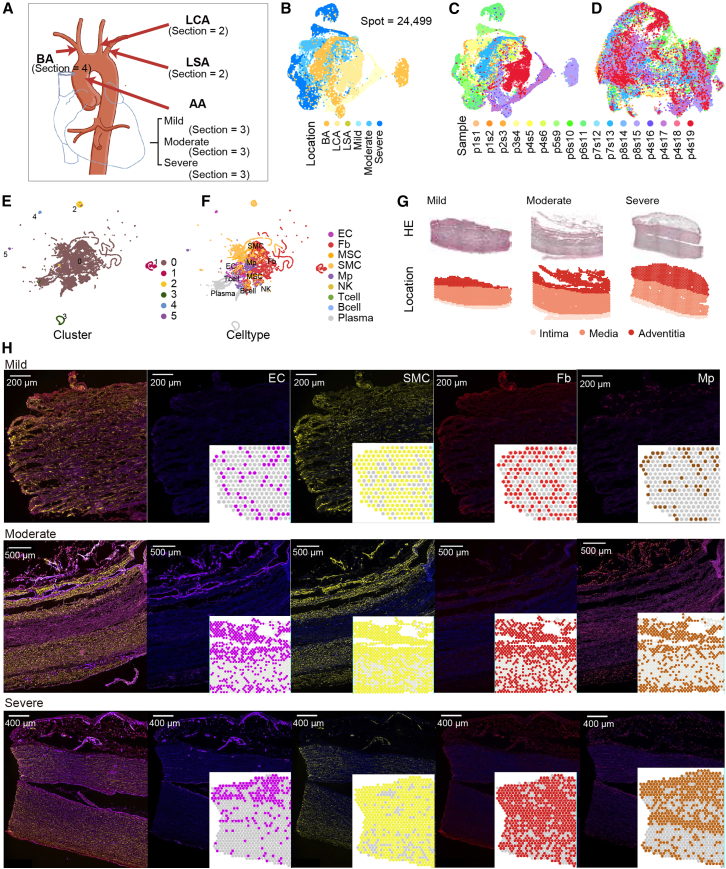
Spatial transcriptomic analysis of AAD tissues (A) Schematic diagram of the aortic sections obtained from patients with AAD. (BA, brachiocephalic artery; LCA, left common carotid artery; LSA, left subclavian artery; AA, ascending aorta). (B) UMAP plot of 24,499 spots across different aortic sections, color-coded by anatomical location and clinical severity. (C) UMAP visualization of harmonized data from all sections, with each color representing a unique patient sample. (D) UMAP visualization of harmonized data from all sections, with each color representing distinct sections. (E) UMAP visualization identifying distinct spot clusters (clusters 0–5) based on spatial gene expression. (F) UMAP plot depicting the annotation of spots into specific cell types. (G) Representative hematoxylin and eosin (H&E) staining (top) and corresponding spatial mapping (bottom) of the distinct anatomical layers (intima, media, and adventitia) across mild, moderate, and severe AAD lesions. (H) mIF validation of the main cell types and their spatial spot arrangements across different severity levels. The specific markers include CD31 (EC marker) in magenta, ACTA2 (SMC marker) in yellow, CALD1 (Fb marker) in red, and CD163 (Mp marker) in brown. Nuclei are counterstained with DAPI (blue). Scale bars are indicated in the respective images (200 μm, 400 μm, and 500 μm).

To conduct this study, 8 patients with a diagnosis of AAD were enrolled and underwent surgical myectomy, and a total of 19 tissue sections were sliced and subjected to spatial transcriptomics analysis (Table 1). There were 11 sections from the ascending aorta (AA), two sections from the left subclavian artery (LSA), two from the left carotid artery (LCA), and four from the brachiocephalic artery (BA). Among them, patient#4 had the lesions in the ascending aorta, left subclavian artery, and left carotid artery, and patients #7 and #8, respectively, had the lesions in the brachiocephalic artery. The rest of the patients had lesions in the ascending aorta. The ascending aorta sections were further divided into three groups to represent the severity levels according to the widest diameter of the ascending aorta (mild, 36–40 mm; moderate, 41–45 mm; and severe, 46–50 mm), as recommended by Sharif et al.^18^^,^^19^^,^^20^ (Figure 1A; Table 1).

**Table 1:** Patient information

Patient	Sex/age	Diagnosis	Additional diagnosis	Anatomical position	WD of AA (mm)	Section	Number of spots under tissue	Number of reads(∗10^6^)	Total genes detected	Median genes per spot	Mean reads per spot
1	M/49	AAD	Primary hypertension	AA	37.6	1	1,274	506.74	18,838	1,809	397,135
						2	1,298	364.25	19,238	1,712	390,103
						18	1,310	364.25	19,238	1,712	267,829
						19	1,299	506.74	19,034	1,628	278,051
2	M/45	AAD	Coronary artery stenosis	AA	39.4	3	848	401.95	16,773	771	164,061
3	M/52	AAD	MOF	AA	41.5	4	2,442	401.95	20,294	1,108	473,439
4	M/56	AAD	MODS	AA	42.8	5	990	480.28	17,970	767	472,716
						6	1,004	480.28	18,042	769	480,280
				LSA	/	14	1,044	448.64	19,002	1,286	428,498
						15	1,063	448.64	19,119	1,398	421,256
				LCA	/	16	1,561	490.73	20,313	2,005	313,767
						17	1,614	490.73	20,184	1,994	303,670
5	M/50	AAD	NYHA class II	AA	46.5	7	1,865	339.19	19,879	1,398	181,097
6	M/52	AAD	MOF	AA	47.5	8	1,743	389.57	20,276	1,667	127,220
						9	1,668	213.98	18,901	1,147	222,357
7	M/53	AAD	Dissection of the aortic arch	BA	/	10	1,213	522.55	17,837	1,208	429,725
						11	1,210	522.55	16,970	982	427,615
8	F/48	AAD	Hypertension class 3	BA	/	12	1,782	366.40	20,610	1,412	109,439
						13	1,880	399.02	20,440	1,357	115,256

Following sequencing and the bioinformatic pipeline in a previous study,^21^ a total of 24,499 spots were identified, with each section having 848–2,442 spots, and for each spot, the median number of detected genes ranged from 767 to 2,005 (Table 1). Data visualization was performed using uniform manifold approximation and projection (UMAP) (Figures 1B–1F), while the t-distributed stochastic neighbor embedding (t-SNE) is provided in the Figure S1. Before batch effect correction, it was obvious that data from each section and each patient were grouped together, indicative of individual specificity (Figures 1B and 1C). To eliminate these differences, Harmony was applied to merge all sections together, displaying the integrated data distribution across different samples and conditions (Figure 1D). Then clustering of deconvolution methods^22^ and annotation with single-cell data^23^ was conducted, and we further displayed all data by six clusters (Figure 1D) and nine major cell types, including endothelial cells (ECs), fibroblasts, mesenchymal stem cells (MSCs), SMCs (SMCs), macrophages, natural killer cells (NKs), T cells, B cells, and plasma (Figure 1F). Meanwhile, visualization of spatial transcriptomic data displayed intima, media, and adventitia according to the HE results (Figure 1G).

To validate the ST findings, mIF was employed to characterize the cellular architecture within the aortic wall at a higher resolution (Figure S2). As AD progressed from mild to severe, we observed a significant increase in macrophage infiltration and a concomitant reduction in SMC markers within the medial layer (all *p* < 0.05). Low-magnification images in Figure 1H provide a comprehensive view of the vascular layers (intima, media, and adventitia), directly corresponding to the spatial distribution of spots identified in the ST data. The high consistency between the ST visualization and the quantitative mIF results reinforces the reliability of our spatial transcriptomic atlas and confirms the dramatic remodeling of the aortic immune-vascular microenvironment.

### Cell-type specific roles in AA dissection

**Figure 2:**
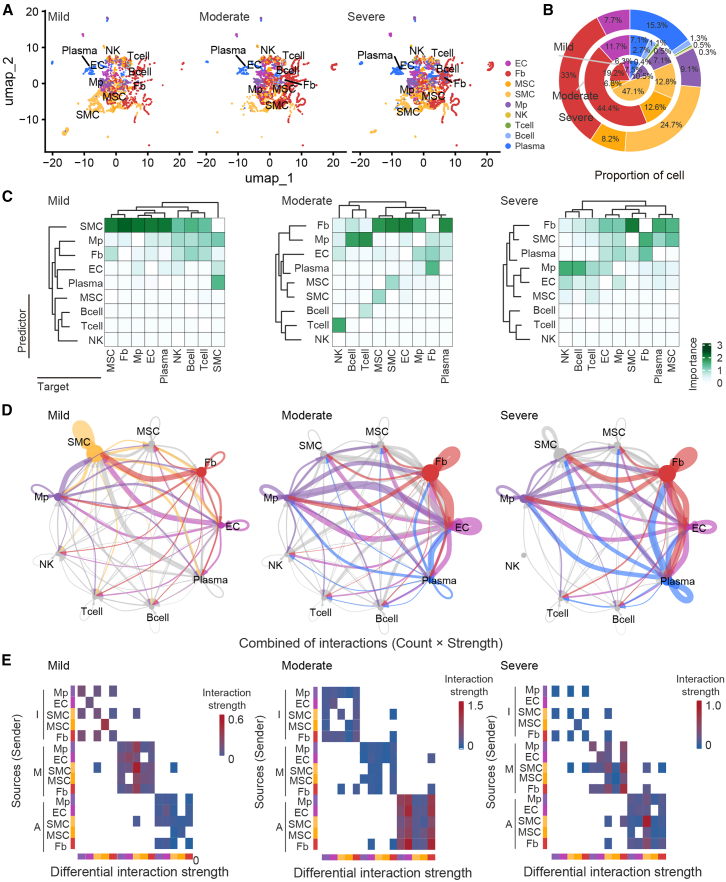
Spatial distribution and inferred cellular interactions across AAD severities (A) UMAP plots illustrating the spatial distribution of diverse cell types across mild, moderate, and severe AAD. (B) Pie chart depicting the relative proportion of each deconvoluted cell type within the ascending aorta across mild (inner ring), moderate (middle ring), and severe (outer ring) AAD. (C) Spatial co-localization heatmaps showing the median predictive importance of a specific cell type’s abundance (Predictor, *y* axis) in determining the abundance of other cell types (Target, *x* axis) within the same spatial spot across disease severities. (D) Network diagrams illustrating the inferred cellular communications (combined count and strength) between different cell types in mild, moderate, and severe AAD. The thickness of the lines indicates the interaction strength, calculated based on ligand-receptor co-expression. (E) Heatmaps detailing the spatial differential interaction strength between source (sender) and target (receiver) cell types across mild, moderate, and severe AAD. The rows denote the sender cells stratified by distinct anatomical layers (I, intima; M, media; A, adventitia). In the color bar, red indicates stronger inferred interactions, while blue indicates weaker or absent interactions.

We first compared the differences in the ascending aorta sections of varied severities using UMAP (Figure 2A). To rigorously evaluate the tissue composition during AD progression, we calculated the relative contribution of each cell type (Table S1; Figure S3A). As shown in the Figure 2B, the inner to outer rings represent the mild, moderate, and severe groups, respectively, providing a holistic comparison of cellular trends. In the mild AA sections, SMCs were notably predominant, comprising approximately 47.1% of the total composition (Figures 2A and 2B). In contrast, the moderate AA section exhibited a shift toward a fibroblast-dominant profile (44.4%), while in the severe AA section, fibroblasts (33%) and SMCs (24.7%) remained the major populations. The overall proportion of immune cells across different severities ranged from 18.5% to 26.5% (Figure 2B; Figure S3B).

We next inferred the contexts among cell types by testing whether the abundance of cell type within spots could be predicted by their spatial context described by the cell-type compositions of their neighborhood. We evaluated three different neighborhood area sizes using MISTy: (1) the importance of cell-type abundances within a spot (colocalization), (2) in the local neighborhood (radius of 2spots), and (3) in an extended neighborhood that expanded to a radius of five spots. In the mild AA dissection, we observed that SMCs were the most predictive of the abundance of structural cell types (MSC, Fb, and EC) and the immuno cell types(Mp, NK, T cell, and B cell), while macrophages showed some importance of predictive of immuno cell types (NK, T cell and B cell)and SMCs (C). This can be explained by the fact that SMCs still formed the main structure of the vessel, and macrophages, possibly the main immune cell type to be involved in the mild form of AA dissection. However, in the moderate AA dissection, fibroblasts became the most predictive of the abundance of the structural cell types (MSC, SMC, and EC) and the estimated importance of macrophages predictive of immuno cell types was increased;and in the severe AA section, fibroblasts and SMCs together formed the majority of predictive of the structural cell types, while macrophages still showed the importance with immuno cell types (Figure 2C). Macrophages and NK, T cell, and B cell showed strong dependencies with each other,in line with immune profiles in moderate and severe AA, similarly captured by mild AA dissection. In some aspects, this reflected the importance of varied cell types in different severities of AA dissection and showed that the fibroblasts became more important in the most severe disease.

Following cell-cell communication networks by using CellPhoneDB were performed (Figure 2D), In mild AAD, the communication network was characterized by strong signals between SMCs and ECs. In more advanced stages, inferred interactions involving fibroblasts became more prominent (Figure 2D). EC connections remained important in the moderate and severe AA dissections. We next separated the interaction strength among cell types across varied morphological layers (Figure 2E). The heatmap reveals an interaction pattern in which nearly all interactions were within the same layer (Figure 2E). The mild AA dissection had the strongest interactions in the media, the moderate had them in the adventitia, while in the severe AA dissection, media and adventitia showed similar interaction strengths (Figure 2E).

### Gene expression in AA dissection

**Figure 3:**
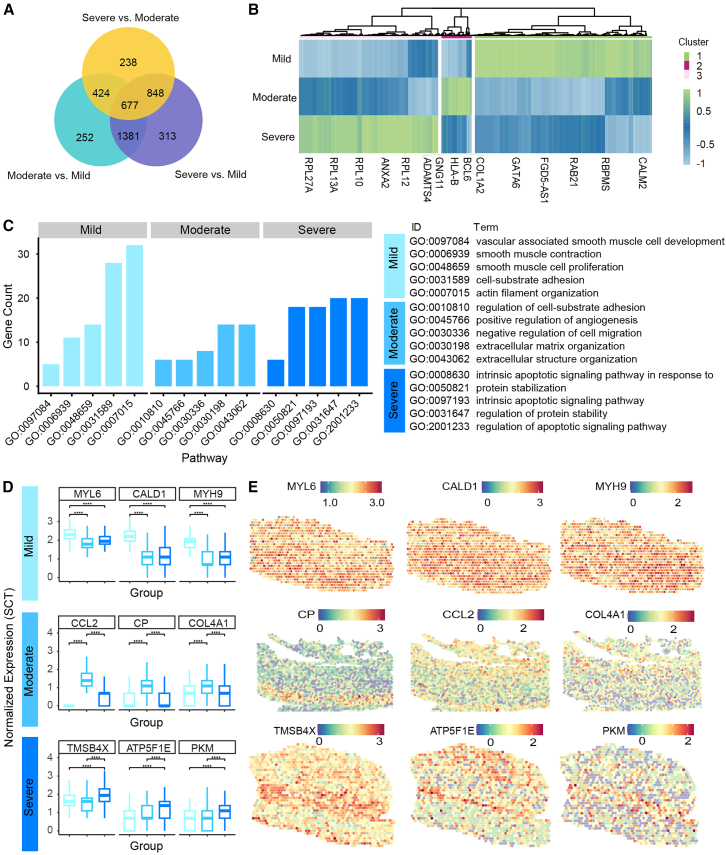
Layer-anchored gene signatures and pathway enrichment across AAD severities (A) Venn diagram depicting the overlap of DEGs among the three severity group comparisons (moderate vs. mild, severe vs. moderate, and severe vs. mild). (B) Heatmap representing the expression patterns of top DEGs grouped into three distinct clusters across mild, moderate, and severe AAD. The color gradient from dark blue to green/yellow indicates low to high scaled expression levels, respectively. (C) Bar graphs illustrating the gene counts for significantly enriched GO pathways specific to mild, moderate, and severe AAD. The corresponding GO terms and IDs are listed on the right. (D) Boxplots showing the normalized expression levels (SCT) of the identified 9-gene severity scale, distinctly categorized into mild (*MYL6*, *CALD1*, *MYH9*), moderate (*CCL2*, *CP*, *COL4A1*), and severe (*TMSB4X*, *ATP5F1E*, *PKM*) progression stages. The central line in each box represents the median, while the lower and upper hinges indicate the 25th and 75th percentiles. ∗∗∗∗*p* < 0.0001. (E) Spatial transcriptomic feature plots demonstrating the corresponding spatial expression patterns of the nine severity-scale marker genes across tissue spots. The color gradient from blue to red represents low to high normalized expression levels.

To elucidate the underlying core signatures contributing to different severities of AA dissection, we conducted a spatial analysis of tissue features based on gene expression. We identified 677 common differentially expressed genes (DEGs) across the three groups, 1,381 specific genes in mild AA dissection, 424 specific genes in moderate AA dissection, and 848 specific genes in severe AA dissection. The significant overlaps among these gene sets highlight both commonalities and differences in the molecular profiles across AA dissection severities (Figure 3A). We assigned putative biological characterizations to each cluster based on gene expression patterns of established canonical markers associated with disease severity (Figure 3B). Cluster 1 was enriched for several genes, including *CALM2*, *RBPMS*, *RAB21*, *FGD5-AS1*, and *GATA6*, which were more highly expressed in mild AA dissection. Cluster 2 was enriched for five genes in moderate AA dissection, such as *COL1A2*, *BCL6*, *HLA-B*, *GNG11*, and *ADAMTS4*. Other genes enriched in the severe AA dissection signature included *RPL12*, *ANXA2*, *RPL10*, *RPL13A*, and *RPL27A*. We further performed GO analysis to explore the key biological processes and pathways associated with different severities of AA dissection (Figure 3C; Table S2). In mild AA dissection, enriched GO terms included smooth muscle contraction, cell-substrate adhesion, and actin filament organization, suggesting early perturbation in vascular homeostasis. In moderate AA dissection, enriched biological processes were generally involved in active tissue remodeling and ECM dynamics. Severe AA dissection was characterized by pathways such as GO:0050821 (protein stabilization), GO:2001233 (regulation of apoptotic signaling pathway), GO:0097193 (intrinsic apoptotic signaling pathway), GO:0031647 (regulation of protein stability), and GO:0008630 (intrinsic apoptotic signaling pathway in response to DNA damage). These pathways are associated with cell apoptosis, indicating a phase of advanced tissue degeneration and immune activation. Subsequently, we focused on specific spatial gene expression patterns for select genes from the top 30across the AA dissection (Figure 3D). Based on the combined criteria of (i) highest transcript abundance within each severity group and (ii) the most stereotyped, layer-restricted spatial distribution, we nominate the following nine genes as putative severity-specific markers for AA dissection: *MYL6*, *CALD1* and *MYH9* for mild AA dissection; *CCL2*, *CP* and *COL4A1* for moderate AA dissection and *TMSB4X*, *ATP5F1E* and *PKM* for severe AA dissection. In mild AA dissection, *MYL6*, *CALD1*, and *MYH9* distribute a full layer consistent with a global but still sub-critical contractile stress response (Figure 3E, upper row). When the disease advances to moderate, the inflammatory-ECM triad (*CCL2*, *CP*, and *COL4A1*) abandons the intima and becomes sharply concentrated in the media, colocalizing precisely with the earliest lamellar separation and micro-hemorrhage (Figure 3E, middle row). In severe AA dissection, the metabolic-stress module (*TMSB4X*, *ATP5F1E*, and *PKM*) forms a narrow, high-intensity band that lines the jagged margins of the medial tear at the interface with the false lumen, demarcating the zone of active structural failure (Figure 3E, lower row). Thus, each severity stage is defined by a unique molecule whose spatial confinement mirrors the evolving mechanical insult. To validate the robustness of our 9-gene severity scoring system, we performed external validation using the publicly available GSE52093 dataset (*n* = 7 AAD patients vs. *n* = 5 controls).^24^ Seven of nine genes (77.8%) were successfully matched and showed biologically consistent expression patterns (Figure S4; Table S3). Notably, mild AA markers (MYL6, ACTB, VIM) exhibited lower expression in AAD patients compared to controls, supporting their interpretation as protective or homeostatic genes whose downregulation may contribute to disease initiation. Conversely, moderate AA markers (CCL2, TMSB4X, FN1, IGFBP7) demonstrated significantly elevated expression in AAD patients, confirming their association with inflammatory responses and ECM remodeling.

### The *SPP1* signaling pathway is a keystone in AAD progression

**Figure 4:**
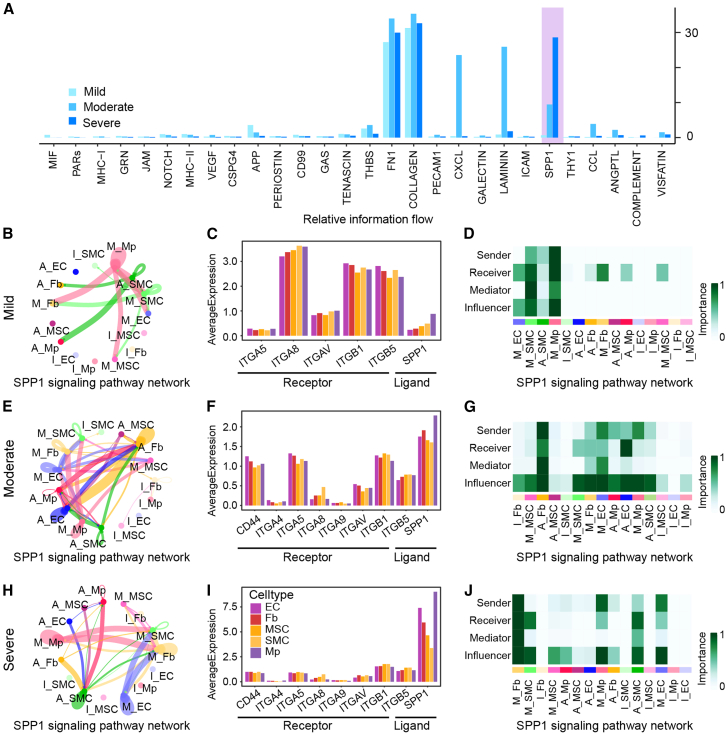
Inferred *SPP1* signaling pathway dynamics across AAD severities (A) Bar graph displaying the relative information flow of various inferred signaling pathways across mild, moderate, and severe AAD. The *x* axis lists specific pathways, with the *SPP1* signaling pathway (highlighted in the purple box) demonstrating a prominent increase in information flow correlating with disease severity. (B–J) Circle plots illustrating the inferred *SPP1* signaling pathway interaction networks among distinct layer-anchored cell types (I, intima; M, media; A, adventitia) in mild (B), moderate (E), and severe (H) AAD lesions. Edge thickness reflects the inferred interaction strength. (C, F, and I) Bar graphs detailing the average expression levels of the *SPP1* ligand and its corresponding receptors (e.g., *CD44*, *ITGA5*, *ITGB1*, *ITGB5*) across major cell types (EC, Fb, MSC, SMC, Mp) in mild (C), moderate (F), and severe (I) AAD. (D, G, J) Heatmaps evaluating the relative importance (network centrality) of different layer-specific cell types acting as senders, receivers, mediators, or influencers within the *SPP1* signaling network across mild (D), moderate (G), and severe (J) AAD. The color gradient from white to dark green represents the importance score (0–1).

To investigate the underlying pathways contributing to the varying severities of AAD, we conducted an analysis of signaling pathways based on gene expression profiles. The bar graph revealed that the expression levels of *FN1* and *COLLAGEN* were upregulated across the disease progression. Interestingly, the *SPP1* pathway exhibited a significant and gradual upregulation with increasing AAD severity (Figure 4A). This suggests that *SPP1* may play a crucial role in the pathophysiology of AAD. To further explore the interactions of different tunica cells in the *SPP1* signaling pathway, we identified frequent cell interactions in mild, moderate, and severe AAD, respectively (Figures 4B–4E, and 4H). Cellular interactions via *SPP1* signaling between media macrophages and other cells were observed across different AAD severities, but these interactions were significantly strengthened in severe AAD, particularly among media macrophages, SMCs, and fibroblasts. This differential interaction of *SPP1* signaling between macrophages and other cells in varying AAD severities may be largely attributed to the differential expression of *SPP1* ligands or receptors in different cell types. In moderate and severe AAD, macrophages expressed high levels of *SPP1* ligands (Figures 4C–4F, and 4I). It is conceivable that communication via *SPP1* signaling between macrophages and other cells was relatively sparse in mild AAD due to the lack of abundant *SPP1* ligand stimulation. However, in moderate and severe AAD, the levels of *SPP1* ligands were widely upregulated in both macrophages and other cells, suggesting that both autocrine and paracrine mechanisms may simultaneously promote constitutive communication of *SPP1* signaling between macrophages and other cells in mild AAD. This differential interaction may be associated with the complex and sometimes paradoxical role of *SPP1* signaling in AAD: in mild AAD, *SPP1* inhibits inflammation, while in moderate and severe AAD, *SPP1* promotes inflammatory responses. We found that in mild AAD, media macrophages act as signal senders, and the receiver participates in the *SPP1* signaling pathway. In moderate AAD, the signal sender was adventitia fibroblasts, with adventitia ECs acting as receivers. In severe AAD, both the signal sender and receiver were media fibroblasts. Regardless of the severity of AAD, media macrophages consistently acted as influencers participating in the *SPP1* signaling pathway (Figures 4D–4G, and 4J). To further validate these findings at the tissue spatial level, we quantified *SPP1* expression across all spatial spots in our samples. SPP1 showed striking progressive upregulation from mild to severe AAD, with a 4.1-fold increase in mean expression (*p* < 0.0001, Figures S5A and S5B). Spatial overlay mapping confirmed that *SPP1*-high regions visually overlapped with CD163-enriched areas, particularly within the medial layer of severe AAD, where SPP1^+^CD163^+^ double-positive spots were substantially enriched (Figure S5C). Spot-level correlation analysis between *SPP1* and the macrophage marker *CD163* revealed progressively stronger spatial co-localization with disease severity (mild: r = 0.041; moderate: r = 0.088; severe: r = 0.287, Figure S5D). These findings suggest that macrophages are a major source of SPP1 ligands, which may contribute to the inflammatory remodeling observed in advanced stages.

### Spatial transcriptional features in AAD

**Figure 5:**
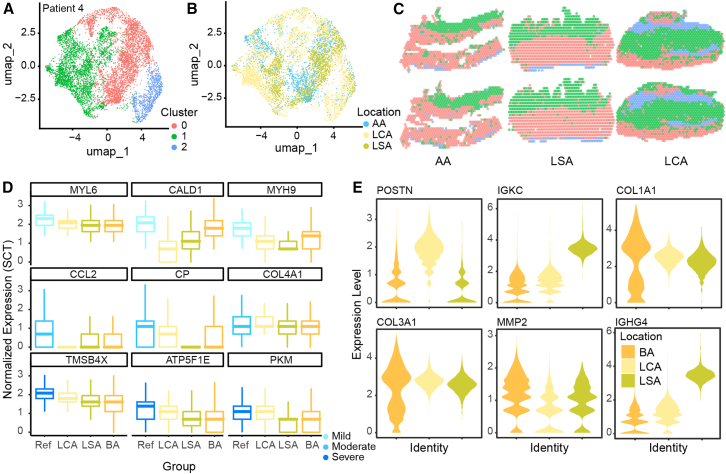
Transcriptomic and spatial heterogeneity across distinct aortic regions (A) UMAP visualization of spatial spots from patient 4, illustrating the distribution of three distinct transcriptional clusters (clusters 0, 1, and 2). (B) UMAP plot of the same spots, color-coded by their anatomical origins: AA, LCA, and LSA. (C) Spatial transcriptomic mapping demonstrating the distribution of the identified clusters (from A) across the corresponding intact tissue sections of the AA, LSA, and LCA. (D) Boxplots showing the normalized expression levels (SCT) of the 9-gene severity scale (*MYL6*, *CALD1*, *MYH9*, *CCL2*, *CP*, *COL4A1*, *TMSB4X*, *ATP5F1E*, *PKM*) across the reference ascending aorta (Ref) and other supra-aortic branches (LCA, LSA, BA). (E) Violin plots detailing the specific expression profiles of the collagen-remodeling triad (*COL1A1*, *COL3A1*, *MMP2*) along with other key genes (*POSTN*, *IGKC*, *IGHG4*) within the different supra-aortic branches (BA, LCA, LSA).

To interrogate whether spatial transcriptional programmes are conserved across distinct aortic segments, we pooled all spots obtained from three anatomical sites of Patient #4 (AA; LCA; LSA) and subjected them to unsupervised UMAP. Three transcriptionally defined clusters emerged (Figure 5A); notably, these clusters did not segregate by sampling location (Figure 5B). Instead, when clusters were mapped back onto the histological architecture, they aligned precisely with the intima, media, and adventitial layers, respectively (Figure 5C), demonstrating that transcriptional identity is layer-defined rather than position-dependent and that equivalent tunicae are directly comparable between aorta segments. We next investigated whether severity-associated gene signatures derived from AA dissections are recapitulated in other segments. After between-sample normalization, the nine severity-specific marker genes calibrated in AA (*MYL6*, *CALD1*, *MYH9* for mild; *CCL2*, *CP*, *COL4A1* for moderate; *TMSB4X*, *ATP5F1E*, *PKM* for severe) were projected onto LCA, LSA, and BA datasets (Figure 5D; Figures S6A–S6C). Surprisingly, LCA and LSA spots exhibited expression intensities most congruent with the mild-AA signature, whereas BA profiles spanned mild-to-moderate ranges, independent of the concurrent AA severities. These observations imply that local mural severity in branch vessels is not a passive mirror of AA status but rather reflects individual-specific, segment-autonomous pathobiology. Inspection of extended spatial maps (Figure S6D) further revealed six genes—*COL1A1*, *COL3A1*, and *MMP2* in addition to the core panel—that display concordant up-regulation across all three sites irrespective of AA severities (Figure 5E). Given their ECM-centric functions and uniform spatial deployment, we posit that *COL1A1*, *COL3A1*, and *MMP2* constitute a conserved “branch-vessel lesion signature” that may serve as sensitive, location-agnostic biomarkers for early dissection-prone remodeling within the aortic arch branches.

## Discussion

AAD is a rapidly progressing cardiovascular disease with a high mortality rate, and its exact pathogenesis remains unclear. The application of spatial transcriptomics and bioinformatics has advanced our ability to gain profound insights into the cellular composition and spatial organization within vascular tissues. Our study delves into the complexity of AAD, with a particular emphasis on the crucial role of cellular and layer-specific gene signatures during pathological progression. The distribution patterns of these cells aligned well with their known functional roles. Notably, we observed a more prominent distribution of ECs in the adventitia with increasing AAD severity. Rather than implying a direct migration of ECs from the intima, this distribution likely reflects localized angiogenesis associated with the vasa vasorum or lymphatic vessel expansion. An important finding in our study is the increased proportion of macrophages which predominantly localized in the intima and near the dissection tears, correlating positively with the severity of AAD. Additionally, the *SPP1* pathway exhibited a significant and gradual upregulation with disease progression. Our data suggest that inferred interactions via *SPP1* signaling play a pivotal role in promoting macrophage activity, highlighting its essential contribution to AAD progression.

The need for complementary studies in the field is further highlighted when comparing our results to previous single-cell RNA sequencing (scRNA-seq) studies that addressed cellular heterogeneity in the normal and dissected ascending aorta.^25^ Using spatial transcriptomics, our study comprehensively assessed the cellular composition and abundance of different cell types across various severity levels while preserving their vital morphological context. We found that the proportion of fibroblasts significantly increased, actively contributing to tissue remodeling and disease progression. While our spatial findings point toward a predominantly pro-inflammatory microenvironment in advanced severities—evidenced by robust macrophage infiltration and inflammatory signaling—further single-cell resolution studies are required to explicitly distinguish between pro- and anti-inflammatory macrophage polarization states and their distinct localizations in these tissues. Nevertheless, our findings strongly align with previous studies underscoring the critical roles of fibroblast-driven tissue remodeling and macrophage-mediated inflammation in AAD.^26^

Our spatial transcriptomic analysis identified distinct gene expression patterns across different aortic regions and layers. Previous studies have identified hub genes such as *CDK2*, *EIF4A1*, *GLRX*, *NNMT*, and *SLCO2A1* involved in DNA damage and repair within the aortic wall of AAD patients, as well as the regulatory role of miR-126 on *ADAM9* expression in aneurysms.^27^^,^^28^ Building upon this, our analysis nominates a severity-associated gene signature (including *MYL6*, *CCL2*, *TMSB4X*, among others). While these site-agnostic molecular signatures offer valuable insights into local mural severities and hold translational potential, they currently serve as candidate markers. Future research must focus on rigorously validating these severity-specific genes using independent clinical cohorts and multi-omics approaches. Confirming their expression at the protein level via quantitative multiplex immunofluorescence or western blot, alongside functional mechanistic models, is essential before considering their diagnostic utility.

*SPP1* (secreted phosphoprotein 1), also known as osteopontin (OPN), is predominantly expressed by macrophages and plays an important regulatory role in cardiac repair after myocardial injury and pathological remodeling.^29^ This activity is critical for SMC phenotypic transition across the vascular wall, directly implicating *SPP1* in the inflammatory response.^30^ Interestingly, *SPP1* spatial expression was found to be significantly higher and closely co-localized with macrophage markers in patients with severe AAD, suggesting its active involvement in disease progression. While previous literature indicates that *SPP1* can upregulate matrix metalloproteinases (MMPs) via the NF-κB signaling pathway—leading to increased tissue degradation—our current spatial dataset could not definitively confirm the specific upregulation of the NF-κB pathway within the SPP1-high clusters.^31^ Clarifying the precise downstream targets of *SPP1* and confirming NF-κB activation in diseased aortic tissues using targeted functional experiments remains a critical priority for future investigations to fully elucidate the SPP1 pathway dynamics in AAD.

Overall, our study provides a spatially resolved transcriptomic landscape of AAD progression, highlighting the gradual upregulation of *SPP1* and its correlation with increasing disease severity. These findings underscore the pivotal role of *SPP1* in promoting pro-inflammatory signaling and macrophage activation within the vascular wall, offering a crucial structural and molecular framework for understanding advanced aortic degeneration.

### Limitations of the study

We acknowledge several important methodological limitations in our study. First, our design lacks a baseline healthy or normal aortic tissue ST control group. Our primary focus, however, was to investigate the spatial heterogeneity within the dissected aorta following disease onset across varying severities. Second, sections dominated by massive intraluminal thrombus were excluded from our baseline spatial analysis to prevent confounding the intrinsic transcriptomic signatures of the aortic wall. Consequently, our study does not fully capture the complex biological interactions at the thrombus-vessel interface, representing another limitation that warrants targeted investigation in future studies. Furthermore, during external validation using the GSE52093 dataset, the absence of detailed clinical severity stratification metadata precluded the direct cross-validation of our severity-dependent scoring gradient, reflecting a prevalent limitation within currently available AAD transcriptomic repositories.

## Resource availability

### Lead contact

Further information and requests for resources and reagents should be directed to and will be fulfilled by the lead contact, Yi-Ning Yang (yangyn5126@163.com).

### Materials availability

This study did not generate new unique reagents.

### Data and code availability

• Spatial transcriptomics sequencing data have been deposited at NCBI Sequence Read Archive (SRA) as BioProject: PRJNA730333 and are publicly available as of the date of publication. Note that while a subset of these data (1 sample) was previously utilized in an earlier study,^21^ the current study comprehensively analyzes all 12 samples to systematically investigate the spatial features in Stanford Type A aortic dissection.
• All original code has been deposited at GitHub and is publicly available at https://github.com/yingcao522/Spatial-Transcriptomics-Analysis-Pipeline as of the date of publication.
• Any additional information required to reanalyze the data reported in this study is available from the lead contact upon request.

## Acknowledgments

This work was financially sponsored by 10.13039/100014718National Natural Science Foundation of China (nos. 82460099, 82570503).

## Author contributions

Conceptualization, Y.-N.Y. and D.L.; methodology, Q.H., Z.L., Y.-H.L., and J.-Y.L.; investigation and data curation, D.A., L.-Y.W., H.-z.L., and Q.Z.; writing – original draft, Y.-H.L. and Y.C.; writing – review and editing, Y.-H.L., Y.C., and F.L.; supervision, Y.-N.Y., D.L., and X.-M.L.; funding acquisition, Y.-H.L. and Y.-N.Y.

## Declaration of interests

The authors declare no competing interests.

## STAR★Methods

### Key resources table

**Table:** 

REAGENT or RESOURCE	SOURCE	IDENTIFIER
**Antibodies**		

Mouse anti-human CD31	Abcam	Cat# 9498
Rabbit anti-human CD163	Abcam	Cat# 182422
Rabbit anti-human CALD1	Abcam	Cat# 32330
Rabbit anti-human HLA-DR	Abcam	Cat# 92511
Rabbit anti-human ACTA2	Abcam	Cat# 124964
Mouse anti-human ELN	Abcam	Cat# 9519
Secondary horseradish peroxidase-conjugated antibody	PerkinElmer	Cat# NEF822001EA

**Biological samples**		

Human AAD tissues (ascending aorta, brachiocephalic artery, left subclavian artery, and left common carotid artery)	Department of Cardiac Surgery, The First Affiliated Hospital of Xinjiang Medical University	N/A

**Chemicals, peptides, and recombinant proteins**		

OCT compound	Tissue-Tek, Sakura Finetek	Cat# 4583
Isopentane (2-methylbutane)	Sigma	Cat# M32631
Methanol (for HPLC, ≥99.9%)	Sigma	Cat# 34860
Isopropanol	Sigma	Cat# I951
Mayer’s Hematoxylin	Sigma	Cat# MHS16
Bluing buffer	Dako	Cat# CS702
Eosin	Sigma	Cat# HT110116
Permeabilization enzyme	10x Genomics	Cat# 1000193
AR9 buffer (pH 6.0)	PerkinElmer	Cat# NEL821001KT
1× amplification diluent	PerkinElmer	Cat# FP1498

**Critical commercial assays**		

EVG Kit	Solarbio	Cat# G1596
Opal 7-Colour Manual IHC Kit	PerkinElmer	Cat# NEL811001KT
Agilent Bioanalyzer High-Sensitivity DNA Kit	Agilent	Cat# 5067-4626

**Deposited data**		

Raw sequencing data	This paper	https://www.ncbi.nlm.nih.gov/sra/PRJNA730333
Analysis code	This paper	https://github.com/yingcao522/Spatial-Transcriptomics-Analysis-Pipeline

**Software and algorithms**		

R software	R Core Team	https://www.r-project.org/; RRID: SCR_001905
Python software (v3.7)	Python Software Foundation	https://www.python.org/; RRID: SCR_008394
bcl2fastq2 (v2.20)	Illumina	https://support.illumina.com/; RRID: SCR_015058
Cutadapt (v1.18)	Pomerantz MM^32^	https://doi.org/10.14806/ej.17.1.200; RRID: SCR_011841
Space Ranger pipeline (v1.0.0)	10x Genomics	https://support.10xgenomics.com/; RRID: SCR_023571
Loupe Browser	10x Genomics	https://www.10xgenomics.com/; RRID: SCR_018555
HISAT2 (v2.0.5)	Daehwan K et al.^33^	https://doi.org/10.1038/s41587-019-0201-4; RRID: SCR_015530
Seurat package (v3.1.3)	Li Y-H et al.^21^	https://satijalab.org/seurat/; RRID: SCR_007322
SeuratData package	Satija Lab	https://github.com/satijalab/seurat-data
RCTD (Robust cell-type decomposition)	Dylan M C et al.^34^	https://doi.org/10.1038/s41587-021-00830-w; RRID: SCR_023249
CellPhoneDB	Yunfang W et al.^35^	https://www.cellphonedb.org/; RRID: SCR_017054
mistyR (v1.2.1)	Jovan T et al.^36^	https://doi.org/10.1093/bioinformatics/btab576; Bioconductor
irGSEA (v1.1.2)	Xinyi G et al.^37^	https://github.com/chuiplusplus/irGSEA
in-form advanced image analysis software (v2.3)	Akoya Biosciences	https://www.akoyabio.com/; RRID: SCR_019155

**Other**		

Adhesion microscope slides	CITOGLAS	Cat#188105
Leica CM1950 cryostat	Leica Biosystems	Cat# 14047643598; RRID: SCR_016831
Leica SCN 400 slide scanner	Leica Biosystems	RRID: SCR_024422
Thermal Cycler	Bio-Rad	Cat# 1861096
Qubit 4	Thermo Fisher Scientific	Cat# Q33238; RRID: SCR_018095
Illumina Nova 6000 platform	Illumina	RRID: SCR_016387
Mantra Quantitative Pathology Workstation	Akoya Biosciences	RRID: SCR_019156

### Experimental model and study participant details

#### Human study participants and aortic tissue samples

A total of 15 patients diagnosed with AAD were initially screened at the Department of Cardiac Surgery within the First Affiliated Hospital of Xinjiang Medical University (Urumqi, China). Following a rigorous screening pipeline based on predefined inclusion and exclusion criteria, 8 patients were formally enrolled in this study. The inclusion criteria required a confirmed clinical and pathological diagnosis of sporadic AAD.^23^ Patients presenting with heritable or syndromic forms of aortopathy (e.g., Marfan syndrome, Loeys-Dietz syndrome), those with a known first-degree relative history of thoracic aortic aneurysms or dissections (TAAD), or individuals whose aortic dissection was secondary to infection, aortitis, trauma, or an isolated pseudoaneurysm were strictly excluded.

A total of 15 human aortic tissue samples were surgically harvested from targeted anatomical locations, including the ascending aorta, brachiocephalic artery, left subclavian artery, and left common carotid artery. Of these, 8 core representative blocks from the enrolled patients met the sample quality thresholds for downstream spatial transcriptomics (ST) profiling. This final cohort comprised 5 ascending aorta samples, 2 brachiocephalic artery samples, and 1 continuous multi-vessel tissue block containing the ascending aorta, left subclavian artery, and left common carotid artery. Based on the maximum diameter of the ascending aorta at the widest segment, samples were stratified into three progressive phases of disease severity: mild (36–40 mm), moderate (41–45 mm), and severe (46–50 mm).

From these 8 patient-derived tissues, a total of 19 coronal sections were successfully positioned onto ST arrays. To prevent confounding the intrinsic mural architectural transcriptomic signatures, 2 specific tissue sections from Patient #1 were excluded from the baseline spatial clustering workflows due to being heavily obscured by massive intraluminal thrombi; however, the critical pathophysiological role of false lumen thrombosis in AAD progression is acknowledged.

Sex and Gender Reporting: Demographics regarding the age, sex, gender, and ancestry/ethnicity of all human participants are systematically compiled in Table 1. Due to the restricted sample size within each stratified disease severity stage, the specific influence or association of sex and gender on the spatial transcriptomic landscapes was not statistically evaluated, which represents a recognized limitation of this study.

All clinical procedures and tissue collections strictly adhered to the ethical principles outlined in the Declaration of Helsinki. Prior to surgical intervention and enrollment, all participants or their legally authorized representatives provided written informed consent, granting explicit permission for tissue utilization and the collection of de-identified clinical metadata. This study received formal institutional oversight and was approved by the Ethics Committee of the First Affiliated Hospital of Xinjiang Medical University under the institutional review board approval number 20150006-8.

#### Human aorta tissue processing and cryosectioning

Fresh human aortic specimens were isolated and processed within 30 min post-excision to maximize RNA integrity and prevent tissue degradation. During initial macroscopic preparation, perivascular adipose tissue (PVAT) and loose excess adventitial matrix were meticulously dissected away using sterile instruments to ensure a strict focus on the vascular mural layers (intima, media, and adventitia). The isolated vascular walls were thoroughly rinsed a minimum of five times with ice-cold sterile saline solution to remove residual luminal blood.

Under sterile conditions on a clean Petri dish placed over dry ice, any remaining intraluminal thrombi and redundant extraneous materials were completely cleared using sterile fine tweezers and micro-scissors. Cleaned mural tissues were trimmed into uniform 6 × 6 mm blocks, immediately embedded in Optimal Cutting Temperature (OCT) compound (Tissue-Tek, Sakura Finetek USA, CA), and snap-frozen by immersion in pre-chilled isopentane (2-methylbutane; Sigma-Aldrich, USA) submerged in a liquid nitrogen/dry ice slurry.

To facilitate high-resolution spatial transcriptomics and spatial protein-gene co-localization analyses, the frozen tissue blocks were vertically cryosectioned at a thickness of 10 μm using a Leica CM1950 cryostat (Leica Biosystems, Germany) maintained at an internal chamber temperature of −20°C. These cryosections were systematically captured onto either specialized adhesion microscope slides (CITOGLAS, China) or pre-prepared Codelink-activated microscope glass slides. All sectioned slides were immediately transferred to long-term storage at −80°C until subsequent library preparation and sequencing.

### Method details

#### ST experiments

##### Fixation, staining, and imaging

The sections were carefully placed on the prepared glass slides and incubated at 37 °C for 1 min. After fixation, they were incubated upright in −20°Cmethanol (for HPLC, ≥99.9%)(Sigma, USA) for 30 min. Then, the sections were washed with phosphate-buffered saline (PBS), treated with isopropanol (Sigma, USA) for 1 min, and air-dried. For tissue hematoxylin and eosin (H&E) staining, sections were immersed in Mayer’s Hematoxylin (Sigma, USA) for 7 min, blued using Bluing buffer (Dako, Denmark) for 2 min, and counterstained with eosin (Sigma, USA) for 20 s. Images were captured using a Leica SCN 400 slide scanner (Leica, Germany).

##### Tissue optimisation and permeabilisation

To optimize the tissue for downstream analysis, aorta sections were incubated at 37°Cwith 70 μL of permeabilization enzyme (10x Genomics, USA) per well for varying durations (3, 6, 12, 18, 24, 30 min). Subsequently, the wells were washed with 0.1x SSC buffer. Fluorescent reverse transcription was performed at 53°C for 45 min. A second removal mix was added to each well and incubated at 56°C for 1 h. This step aimed to detach the surface probes. Afterward, 70 μL from each well was removed. Following probe release, hybridization and imaging were performed to detect the features of non-released DNA oligonucleotide fragments and obtain Cy3-images for alignment. The bright-field and fluorescent images were manually aligned using a loupe browser, utilizing visible spots and structures from both images. After fluorescence imaging, the optimal permeabilization time that ensures sufficient mRNA release and minimizes mRNA diffusion during library preparation was selected. Permeabilization was performed as described in the tissue optimization section, skipping tissue removal. The array was printed to include all the necessary sequences for mRNA capture, but without a spatial barcode.

##### Reverse transcription and library construction

To synthesize cDNA, we dispensed 70 μL of reverse transcription reaction mix into each well of the sample section. The cDNA synthesis was carried out using a Thermal Cycler (BIO-RAD, USA) according to the manufacturer’s instructions. Subsequently, second-strand synthesis, cDNA denaturation, cDNA amplification, fragmentation, end repair, A-tailing, adapter ligation, post-ligation purification, and final purification steps were performed using SPRIselect. The cDNA was then amplified by polymerase chain reaction (PCR) using a dual index plate TT Set A well ID and purified using carboxylic acid beads. The size distribution of the final libraries was analyzed using the Agilent Bioanalyzer High-Sensitivity DNA Kit. The concentration of the libraries was measured using Qubit 4 (Thermo Fisher Scientific, USA). Finally, the libraries were subjected to paired-end sequencing on an Illumina Nova 6000 platform. For sequencing, 28bp reads were obtained from read one to determine the spatial barcode and UMI, while 120bp reads were obtained from read two to cover the genetic region.

#### Elastic Van Giessen (EVG) staining

Aortas from humans were embedded in OCT, and 5-μm-thick serial sections were prepared for EVG staining using the EVG Kit (Baso, China) following the manufacturer’s instructions.

#### Multi-color immunofluorescence staining

The specimens were embedded in OCT and sectioned into 8 μm-thick serial cryosections. To address tissue preservation and prevent damage during antigen retrieval, fresh frozen sections were first post-fixed in 4% paraformaldehyde (PFA) for 15 min at room temperature prior to staining. To validate the specific cell types and their spatial distribution, multiplex immunofluorescence (mIHC) was performed using the Opal 7-Colour Manual IHC Kit (PerkinElmer, USA) according to the manufacturer’s protocol, with specific adaptations for sequential multiplexing.

Antigens were retrieved using AR9 buffer (pH 6.0, PerkinElmer, USA) by heating in a microwave oven for 15 min. The multiplex staining involved sequential, repetitive cycles for each target protein. For each cycle, sections were pre-incubated with blocking buffer at room temperature for 10 min, followed by a 1-h incubation at room temperature with one of the following primary antibodies: mouse anti-human CD31 (Abcam, ab9498, UK, 1:200), rabbit anti-human CD163 (Abcam, ab182422, UK, 1:1000), rabbit anti-human CALD1 (Abcam, ab32330, UK, 1:300), rabbit anti-human HLA-DR (Abcam, ab92511, UK, 1:100), rabbit anti-human ACTA2 (Abcam, ab124964, UK, 1:300), or mouse anti-human ELN (Abcam, ab9519, UK, 1:100). A secondary horseradish peroxidase (HRP)-conjugated antibody (PerkinElmer, Waltham, MA, USA) was applied and incubated at room temperature for 10 min. Signal amplification was then performed by incubating the sections with the respective Opal TSA fluorophore (diluted 1:100 in 1× amplification diluent, PerkinElmer, USA) for 10 min at room temperature. After each TSA step, sections were subjected to a microwave heat treatment in AR9 buffer to strip the non-covalently bound primary and secondary antibodies while preserving covalently deposited Opal fluorophores. A negative control slide (incubated with blocking buffer instead of primary antibodies) was included to account for the high autofluorescence of human aortic fibers and red blood cells. Nuclei were counterstained with DAPI.

#### Quantitative analysis of mIF images

To rigorously evaluate spatial distribution and protein validation, multispectral imaging was conducted using a Mantra Quantitative Pathology Workstation (PerkinElmer, CLS140089) at 20× magnification. The captured images were spectrally unmixed and quantitatively analyzed using inForm advanced image analysis software (PerkinElmer, version 2.3). The software was trained to segment DAPI-stained nuclei and appropriately define cytoplasmic/membrane boundaries. Positivity thresholds were established based on negative controls. Statistical comparisons of mIF signals and cell proportions across severity groups were conducted using one-way ANOVA or Student’s *t* test in GraphPad Prism, with *p* < 0.05 considered statistically significant.

#### Reproducibility

All experiments were conducted at least twice, yielding similar results. The number of biological replicates for each experiment is provided individually in the corresponding figure legends.

#### Computational methods

##### Data quality and filtering

The procedures used for processing the original sequencing data were similar to those reported in previous publications from our laboratory.^21^ Briefly, the base call files (BCLs) generated by the Illumina sequencer were first converted to FASTQ format using the Bcl2Fastq2 conversion software (v2.20). The resulting FASTQ files were then subjected to quality control to filter out low-quality reads. The read 1 and read 2 FASTQ files were trimmed to the desired length and quality using Cutadapt (version 1.18)^32^ for subsequent analysis. After selecting the spot loops of interest using Loupe Browser, individual libraries were processed to generate Space feature counts using automatic benchmark alignment and organization detection. Trimmed reads were processed using the Space Ranger pipeline (version 1.0.0). Clean reads were aligned to the human genome (GRCh38 v93) using Hisat2 (version 2.0.5).^33^ Human gene symbols were uniformly capitalized and italicized (e.g., *SPP1*, *MYL6*) throughout the analysis and manuscript.

##### External bulk RNA-seq validation

To validate the translational robustness of the identified 9-gene severity scale (including *MYL6*, *CCL2*, *TMSB4X*, etc.), bulk RNA-seq data from independent AAD cohorts were downloaded from the GSE52093 dataset. Gene expression trends of these markers were cross-validated across the external cohorts using appropriate statistical testing.

##### Normalisation, clustering, and visualisation

Data normalization and variance stabilization were performed using the Seurat package (version 3.1.3) in R (Ma et al., 2018). Based on previous studies,^21^ we determined the key parameters as follows: firstly, the data was standardized using principal component analysis, and the first 30 principal components were used for clustering by the k-nearest Neighbor algorithm (KNN). Next, the distance between each point and other points was calculated, and a shared nearest neighbor (SNN) graph was constructed based on the distance between sample points. Finally, the FindClusters function was applied to identify the cluster. (FindNeighbors settings: reduction = “pca”, nn.method = “rann”, dims = 1:30, k.param = 20; Find Clusters settings: resolution = 0.8, method = “matrix”). To better preserve both local cluster relationships and global tissue architecture, which is critical for spatial transcriptomics, Uniform Manifold Approximation and Projection (UMAP) was employed for dimensionality reduction. Pathologists annotated the 3 layers of arteries based on HE-stained images. We used these annotations to group the spots in the spatial transcriptome data.

##### Identification of cluster-specific genes

For each cluster identified, we determined the differentially expressed genes (DEGs) based on their relationship with all other spots. Initially, we generated a spatial cluster gene list for all genes that were differentially expressed in ST clusters with an average log fold change (logFC) greater than 0.25 and an adjusted *p*-value less than 0.05, and only retained positively regulated genes. The mean expression level of each gene across all spots in the cluster was calculated to identify genes that were enriched in a specific cluster. Next, we compared the expression of each gene from one cluster with the average expression of the same gene from the spots of all other clusters. The DEGs with the largest changes in expression in each cluster were ranked according to their expression differences and were subsequently checked and visualized using heat maps.

##### Spatial deconvolution, colocalization, and niche identification

To resolve the cellular composition within the multi-cellular Visium spots, we performed spatial deconvolution using the Robust Cell Type Decomposition (RCTD) algorithm (v2.0).^34^ We utilized a high-quality single-cell RNA-seq reference dataset^23^ to guide the decomposition. RCTD objects were created for each section, and the pipeline was executed with the following parameters: max_cores = 24, test_mode = FALSE, CELL_MIN_INSTANCE = 6, and doublet_mode = ‘full.’ This yielded normalized weights representing the proportion of each cell type within every individual spot. To determine whether these spots formed organized biological structures rather than random cellular overlaps, we identified cell-type niches following the method described by Kleshchevnikov et al.^38^ Specifically, the cell-type proportions calculated by RCTD for each spot were treated as an n-dimensional vector (where n is the number of cell types) and embedded as a new assay within the Seurat object. Following normalization, unsupervised clustering was performed on these composition vectors to identify groups of spots with similar cellular microenvironments, which we defined as major cell-type niches. These niches were visualized using UMAP (updated from t-SNE) to reflect the spatial transcriptomic landscape. Pie charts representing the deconvoluted proportion of each cell population per spot were generated to distinguish true cellular niches from random cell-type overlap (Figure S3). Additionally, we employed mistyR (v1.2.1)^36^ to estimate the importance of each major cell type’s abundance in explaining the spatial distribution and abundance of others. To validate the spatial co-expression of severity-scale marker genes (e.g., MYL6, CALD1, and MYH9), spatial colocalization analysis was performed. We utilized the squidpy framework in Python to compute spatial neighborhood graphs and conducted permutation testing to quantitatively confirm that these genes colocalize within the same cellular neighborhoods beyond random chance.

##### Identification of cell types

To identify and annotate distinct cellular populations within the spatial transcriptomic data, we employed a comprehensive approach combining unbiased database-driven enrichment with manual curation based on canonical markers. Initially, the CellMarker database^39^ was utilized as a reference. All human cell types and their corresponding marker genes were integrated into a dataset, and cluster-specific genes from our samples were subjected to a hypergeometric test using the enricher function in the clusterProfiler package (version 3.12.0)^40^ with a *p*-value cutoff of 0.005.

To validate and biologically refine these automated annotations, we evaluated the robust expression of established canonical marker genes across the clusters. Specifically, endothelial cells were confirmed by the expression of *PECAM1* and *VWF*; SMCs by *ACTA2*, *MYH11*, and *TAGLN*; fibroblasts by *DCN* and *LUM*; and macrophages by *CD68* and *CD163*. Furthermore, the gene expression matrix of each spot was compared against the Human Cell Landscape (HCL) database using the scHCL function^41^ (settings: numbers_plot = 10) to verify the cell types mapping to different spatial locations. Ultimately, these integrated, marker-based annotations were highly consistent with the specific histological structures observed in the matched H&E and multi-color mIF images, ensuring robust spatial localization.

##### Analysis of cellular communication

In this study, we employed the CellPhoneDB method to compute and infer cell-cell interactions using the Python package.^35^ After further cell type identification for each cluster using the CellMarker and Human Cell Landscape databases, expression profiles and cell grouping files were generated using the SeuratData package. Then, CellPhoneDB was run under Python version 3.7 and graphed (--counts-data = gene_name, --iterations = 1000).

##### Gene set enrichment analysis

Enrichment scores were calculated with the R package irGSEA (version 1.1.2) and the “singscore” method to analyze the differential expression between different severity AAD. We selected the C2 subdatabase of the Molecular Signatures Database (MSigdb) for gene set mapping.^37^ Statistically significant pathways were identified using a *p*-value threshold of less than 0.05. The top 30 pathways were shown.

### Quantification and statistical analysis

For immunofluorescence quantification across disease stages, differences were analyzed using one-way ANOVA followed by Tukey’s post-hoc test, with *p* < 0.05 considered statistically significant. For public dataset validation (GSE52093), data differences were evaluated using the two-tailed unpaired Student’s *t* test, with exact *p*-values indicated in the figures. For spatial expression and co-localization correlation, linear regression and Pearson correlation analyses were adopted, and the correlation coefficient (r) and exact *p*-values were calculated. All statistical analyses and graphical representations in this study were performed using R software.

## Supplemental information

Document S1. Figures S1–S6

Table S1. Cell type contribution

Table S2. Significantly enriched gene ontology (GO) pathways and associated gene signatures across AAD severities

Table S3. Differential expression analysis of the severity-associated gene signature in the external GSE52093 Cohort

## References

[1] Yang K., Ren J., Li X., Wang Z., Xue L., Cui S., Sang W., Xu T., Zhang J., Yu J. (2020). Prevention of aortic dissection and aneurysm via an ALDH2-mediated switch in vascular SMC phenotype. Eur. Heart J..

[2] Sowa A.K., Kaiser F.J., Eckhold J., Kessler T., Aherrahrou R., Wrobel S., Kaczmarek P.M., Doehring L., Schunkert H., Erdmann J., Aherrahrou Z. (2013). Functional interaction of osteogenic transcription factors Runx2 and Vdr in transcriptional regulation of Opn during soft tissue calcification. Am. J. Pathol..

[3] Pape L.A., Awais M., Woznicki E.M., Suzuki T., Trimarchi S., Evangelista A., Myrmel T., Larsen M., Harris K.M., Greason K. (2015). Presentation, Diagnosis, and Outcomes of Acute Aortic Dissection: 17-Year Trends From the International Registry of Acute Aortic Dissection. J. Am. Coll. Cardiol..

[4] Xue L., Luo S., Ding H., Liu Y., Huang W., Fan X., Wu M., Jian X., Huang C., Luo J., Fan R. (2019). Upregulation of miR-146a-5p is associated with increased proliferation and migration of vascular SMCs in aortic dissection. J. Clin. Lab. Anal..

[5] Vickovic S., Eraslan G., Salmén F., Klughammer J., Stenbeck L., Schapiro D., Äijö T., Bonneau R., Bergenstråhle L., Navarro J.F. (2019). High-definition spatial transcriptomics for in situ tissue profiling. Nat. Methods.

[6] Rodriques S.G., Stickels R.R., Goeva A., Martin C.A., Murray E., Vanderburg C.R., Welch J., Chen L.M., Chen F., Macosko E.Z. (2019). Slide-seq: A scalable technology for measuring genome-wide expression at high spatial resolution. Science (1979).

[7] Stickels R.R., Murray E., Kumar P., Li J., Marshall J.L., Di Bella D.J., Arlotta P., Macosko E.Z., Chen F. (2021). Highly sensitive spatial transcriptomics at near-cellular resolution with Slide-seqV2. Nat. Biotechnol..

[8] Liu Y., Yang M., Deng Y., Su G., Enninful A., Guo C.C., Tebaldi T., Zhang D., Kim D., Bai Z. (2020). High-Spatial-Resolution Multi-Omics Sequencing via Deterministic Barcoding in Tissue. Cell.

[9] Schordan S., Grisk O., Schordan E., Miehe B., Rumpel E., Endlich K., Giebel J., Endlich N. (2013). OPN deficiency results in severe glomerulosclerosis in uninephrectomized mice. Am. J. Physiol. Renal Physiol..

[10] Moncada R., Barkley D., Wagner F., Chiodin M., Devlin J.C., Baron M., Hajdu C.H., Simeone D.M., Yanai I. (2020). Integrating microarray-based spatial transcriptomics and single-cell RNA-seq reveals tissue architecture in pancreatic ductal adenocarcinomas. Nat. Biotechnol..

[11] Potente M., Mäkinen T. (2017). Vascular heterogeneity and specialization in development and disease. Nat. Rev. Mol. Cell Biol..

[12] Touyz R.M., Alves-Lopes R., Rios F.J., Camargo L.L., Anagnostopoulou A., Arner A., Montezano A.C. (2018). Vascular smooth muscle contraction in hypertension. Cardiovasc. Res..

[13] Zhao G., Lu H., Chang Z., Zhao Y., Zhu T., Chang L., Guo Y., Garcia-Barrio M.T., Chen Y.E., Zhang J. (2020). Single cell RNA sequencing reveals the cellular heterogeneity of aneurysmal infrarenal abdominal aorta. Cardiovasc. Res..

[14] Asp M., Giacomello S., Larsson L., Wu C., Fürth D., Qian X., Wärdell E., Custodio J., Reimegård J., Salmén F. (2019). A Spatiotemporal Organ-Wide Gene Expression and Cell Atlas of the Developing Human Heart. Cell.

[15] Gu W., Ni Z., Tan Y.Q., Deng J., Zhang S.J., Lv Z.C., Wang X.J., Chen T., Zhang Z., Hu Y. (2019). Adventitial Cell Atlas of wt (Wild Type) and ApoE (Apolipoprotein E)-Deficient Mice Defined by Single-Cell RNA Sequencing. Arterioscler. Thromb. Vasc. Biol..

[16] Kalluri A.S., Vellarikkal S.K., Edelman E.R., Nguyen L., Subramanian A., Ellinor P.T., Regev A., Kathiresan S., Gupta R.M. (2019). Single-Cell Analysis of the Normal Mouse Aorta Reveals Functionally Distinct Endothelial Cell Populations. Circulation.

[17] Pedroza A.J., Tashima Y., Shad R., Cheng P., Wirka R., Churovich S., Nakamura K., Yokoyama N., Cui J.Z., Iosef C. (2020). Single-Cell Transcriptomic Profiling of Vascular SMC Phenotype Modulation in Marfan Syndrome Aortic Aneurysm. Arterioscler. Thromb. Vasc. Biol..

[18] Sharif M., Yap Z.J., Ghazal A., Bashir M., Harky A. (2019). Tear Size and Location Influence the Pressure of False Lumen Following Type A Aortic Dissection: Perspective of Current Evidence. Heart Lung Circ..

[19] Booher A.M., Isselbacher E.M., Nienaber C.A., Froehlich J.B., Trimarchi S., Cooper J.V., Demertzis S., Ramanath V.S., Januzzi J.L., Harris K.M. (2011). Ascending thoracic aorta dimension and outcomes in acute type B dissection (from the International Registry of Acute Aortic Dissection [IRAD]). Am. J. Cardiol..

[20] Truong Q.A., Bhatia H.S., Szymonifka J., Zhou Q., Lavender Z., Waxman A.B., Semigran M.J., Malhotra R. (2018). A four-tier classification system of pulmonary artery metrics on computed tomography for the diagnosis and prognosis of pulmonary hypertension. J. Cardiovasc. Comput. Tomogr..

[21] Li Y.-H., Cao Y., Liu F., Zhao Q., Adi D., Huo Q., Liu Z., Luo J.Y., Fang B.B., Tian T. (2021). Visualization and Analysis of Gene Expression in Stanford Type A Aortic Dissection Tissue Section by Spatial Transcriptomics. Front. Genet..

[22] Ma Y., Zhou X. (2022). Spatially informed cell-type deconvolution for spatial transcriptomics. Nat. Biotechnol..

[23] Li Y., Ren P., Dawson A., Vasquez H.G., Ageedi W., Zhang C., Luo W., Chen R., Li Y., Kim S. (2020). Single-Cell Transcriptome Analysis Reveals Dynamic Cell Populations and Differential Gene Expression Patterns in Control and Aneurysmal Human Aortic Tissue. Circulation.

[24] Pan S., Wu D., Teschendorff A.E., Hong T., Wang L., Qian M., Wang C., Wang X. (2014). JAK2-centered interactome hotspot identified by an integrative network algorithm in acute Stanford type A aortic dissection. PLoS One.

[25] He Y.B., Jin H.Z., Zhao J.L., Wang C., Ma W.R., Xing J., Zhang X.B., Zhang Y.Y., Dai H.D., Zhao N.S. (2022). Single-cell transcriptomic analysis reveals differential cell subpopulations and distinct phenotype transition in normal and dissected ascending aorta. Mol. Med..

[26] Liu Y., Zou L., Tang H., Li J., Liu H., Jiang X., Jiang B., Dong Z., Fu W. (2022). Single-Cell Sequencing of Immune Cells in Human Aortic Dissection Tissue Provides Insights Into Immune Cell Heterogeneity. Front. Cardiovasc. Med..

[27] Aifang Z., Yuzhong C., Yang Z., Ning D., Guifang Y., Xiangping C. (2023). Identification and Analysis of Hub Genes and Immune Cells Associated with the Formation of Acute Aortic Dissection. Comput. Math. Methods Med..

[28] Guanghui S., Qingfeng S., Ye Y., Li S., Liu G., Yuan C., Li H., Xu Y., Wang H. (2020). Role of ADAM9 and miR-126 in the development of abdominal aortic aneurysm. Atherosclerosis.

[29] Zhao Y., Huang Z., Gao L., Ma H., Chang R. (2024). Osteopontin/SPP1: a potential mediator between immune cells and vascular calcification. Front. Immunol..

[30] Li G., Zhang Y., Jiang H., Wu X., Hao Y., Su Y., Zou Y., Xian W., Wang F., Du Q. (2025). PPARG/SPP1/CD44 signaling pathway in alveolar macrophages: Mechanisms of lipid dysregulation and therapeutic targets in idiopathic pulmonary fibrosis. Heliyon.

[31] Uhlig M., Billig S., Wienhold J., Schumacher D. (2025). Pro-Fibrotic Macrophage Subtypes: SPP1+ Macrophages as a Key Player and Therapeutic Target in Cardiac Fibrosis?. Cells.

[32] Pomerantz M.M., Li F., Takeda D.Y., Lenci R., Chonkar A., Chabot M., Cejas P., Vazquez F., Cook J., Shivdasani R.A. (2015). The androgen receptor cistrome is extensively reprogrammed in human prostate tumorigenesis. Nat. Genet..

[33] Daehwan K., Joseph M.P., Chanhee P., Christopher B., Steven L.S. (2019). Graph-based genome alignment and genotyping with HISAT2 and HISAT-genotype. Nat. Biotechnol..

[34] Cable D.M., Murray E., Zou L.S., Goeva A., Macosko E.Z., Chen F., Irizarry R.A. (2021). Robust decomposition of cell type mixtures in spatial transcriptomics. Nat. Biotechnol..

[35] Wang Y., Qin J., Wang S., Zhang W., Duan J., Zhang J., Wang X., Yan F., Chang M., Liu X. (2016). Conversion of Human Gastric Epithelial Cells to Multipotent Endodermal Progenitors using Defined Small Molecules. Cell Stem Cell.

[36] Jovan T., Ricardo Omar Ramirez F., Attila G., Denis S., Julio S.-R. (2022). Explainable multiview framework for dissecting spatial relationships from highly multiplexed data. Genome Biol..

[37] Guo X., Zhang Y., Zheng L., Zheng C., Song J., Zhang Q., Kang B., Liu Z., Jin L., Xing R. (2018). Global characterization of T cells in non-small-cell lung cancer by single-cell sequencing. Nat Med.

[38] Kuppe C., Ramirez Flores R.O., Li Z., Hayat S., Levinson R.T., Liao X., Hannani M.T., Tanevski J., Wünnemann F., Nagai J.S. (2022). Spatial multi-omic map of human myocardial infarction. Nature.

[39] Zhang X., Lan Y., Xu J., Quan F., Zhao E., Deng C., Luo T., Xu L., Liao G., Yan M. (2019). CellMarker: a manually curated resource of cell markers in human and mouse. Nucleic Acids Res..

[40] Yu G., Wang L.G., Han Y., He Q.Y. (2012). clusterProfiler: an R package for comparing biological themes among gene clusters. OMICS.

[41] Han X., Zhou Z., Fei L., Sun H., Wang R., Chen Y., Chen H., Wang J., Tang H., Ge W. (2020). Construction of a human cell landscape at single-cell level. Nature.

