# Supplementary material for: Spatial transcriptomic analysis reveals spatial features in human Stanford type A aortic dissection
Source: iScience. 2026 Jul 16;29(8):116868. doi: 10.1016/j.isci.2026.116868 (PMC13383875; doi:10.1016/j.isci.2026.116868)
Supplement: Document S1. Figures S1–S6 [file mmc1.pdf]

iScience, Volume *29*

Supplemental information

Spatial transcriptomic analysis reveals spatial features in human Stanford type A aortic dissection

Yan-Hong Li, Ying Cao, Fen Liu, Li-Ying Wang, Hai-zhou Liu, Qian Zhao, Dilare Adi, Qiang Huo, Zheng Liu, Jun-Yi Luo, Xiao-Mei Li, Di Liu, and Yi-Ning Yang

Supplementary Figures

Figure S1. Spatial transcriptomic data integration and clustering overview
(A) t-SNE plot of 24,499 spatially resolved spots across the different aortic sections, color-coded by their respective anatomical locations.
(B) t-SNE visualization demonstrating the successful integration and harmonization of data across all sections, with each color representing a unique patient sample to confirm the mitigation of batch effects.
(C) t-SNE plot mapping the spots after comprehensive clustering and spatial deconvolution, with colors indicating the distinct tissue sections.
(D) t-SNE visualization identifying the distinct transcriptomic clusters driven by genome-wide spatial gene expression profiles.

(E) t-SNE visualization depicting the specific cell types identified across all spatial spots following cell-type annotation.

Figure S2. Quantitative multiplex immunofluorescence analysis of cellular identities and structural remodeling across AAD severities
(A) Representative high-magnification (20×) multiplex immunofluorescence (mIF) images detailing the cellular identities and extracellular matrix integrity in the ascending aorta across mild, moderate, and severe AAD lesions. The panels display individual and merged channels for distinct structural and phenotypic markers: CD31 (EC marker) in magenta, ACTA2 (SMC marker) in yellow, CALD1 (Fb marker) in red, and CD163 (Mp marker) in brown. HLA-DR (dendritic cells) in light grey, and ELN (elastic fibers) in green. Nuclei were counterstained with DAPI (blue). Scale bars: 50 µm.
(B) Quantitative statistical analysis of the corresponding mIF signals across the three severity groups. Bar graphs illustrate the positive staining proportions (%) of CD31, CALD1, ACTA2, CD163, HLA-DR, and ELN. Data are presented as mean ± SD. Statistical significance was determined using ANOVA, with P-values explicitly indicated as follows: ns, not significant; * $P < 0.05$; ** $P < 0.01$; *** $P < 0.001$; **** $P < 0.0001$.

Figure S3. Quantitative demographics and spatial deconvolution mapping of cell populations

(A) Spatial scatter pie charts illustrating the deconvolution results based on normalized weights. Each pie chart represents an individual spatial spot, detailing the proportional contribution of distinct cell populations within that specific microenvironment.
(B) Quantitative bar graph detailing the absolute number of cells assigned to each identified cell type across the entire spatial transcriptomic dataset.
(C) Stacked bar charts depicting the relative proportions of distinct cell types across

different anatomical regions of the aorta.
(D) Stacked bar charts illustrating the relative cell-type proportions stratified.
(E) Quantitative analysis comparing the absolute cell counts of the identified cell types across varying degrees of AAD severity (mild, moderate, and severe lesions).

Figure S4. External transcriptomic validation of the layer-anchored 9-gene severity signature
External validation of the 9-gene severity signature in independent AAD cohort (GSE52093). Boxplots showing expression levels of 7 successfully matched genes in AAD patients (n=7, red) versus controls (n=5, blue). Mild severity markers (MYL6, ACTB, VIM) show higher expression in controls, consistent with their role as protective/homeostatic genes. Moderate severity markers (CCL2, TMSB4X, FN1, IGFBP7) show elevated expression in AAD, confirming their association with disease-related inflammation and ECM remodeling. P-values were calculated using Student's t-test. (*P < 0.05; **P < 0.01; ***P < 0.001.)

Figure S5. Spatial colocalization of *SPP1* with macrophage marker *CD163* across AAD severities
(A) Bar graph demonstrating the progressive upregulation of SPP1 gene expression across mild, moderate, and severe AAD. Data are presented as mean normalized expression ± standard error (SE).
(B) Spatial transcriptomic feature plots illustrating the spatial distribution and expression intensity of SPP1 (top row) and the macrophage-specific marker CD163 (bottom row) within representative aortic sections across varying disease severities. The color scales reflect normalized expression levels.
(C) Spatial co-localization mapping of SPP1 and CD163 within the tissue microenvironment. Individual spatial spots are color-coded to indicate their expression status: grey (neither gene expressed), orange (SPP1 expression only), light blue (CD163 expression only), and red (co-expression of both SPP1 and CD163). The density of red spots highlights the spatial overlap.
(D) Scatter plots depicting the expression correlation between SPP1 and CD163 across mild, moderate, and severe AAD spots. The solid red line represents the linear regression fit. The correlation coefficient (r) and statistical significance (P-value) are displayed above each plot, demonstrating an increasingly robust positive association parallel to disease severity.

Figure S6 Spatial distribution of differentially expressed genes across aortic regions

(A) Spatial expression patterns of genes specifically upregulated in mild AA across other aortic regions, including the LSA, LCA, and BA, for patients 4, 7, and 8.

(B) Spatial expression patterns of genes specifically downregulated in moderate AA across the LSA, LCA, and BA regions for patients 4, 7, and 8.

(C) Spatial expression patterns of genes specifically upregulated in severe AAD across the LSA, LCA, and BA regions for patients 4, 7, and 8.

(D) Spatial expression patterns of genes that are commonly expressed across different aortic regions (LSA, LCA, BA), irrespective of disease severity, for patients 4, 7, and 8.

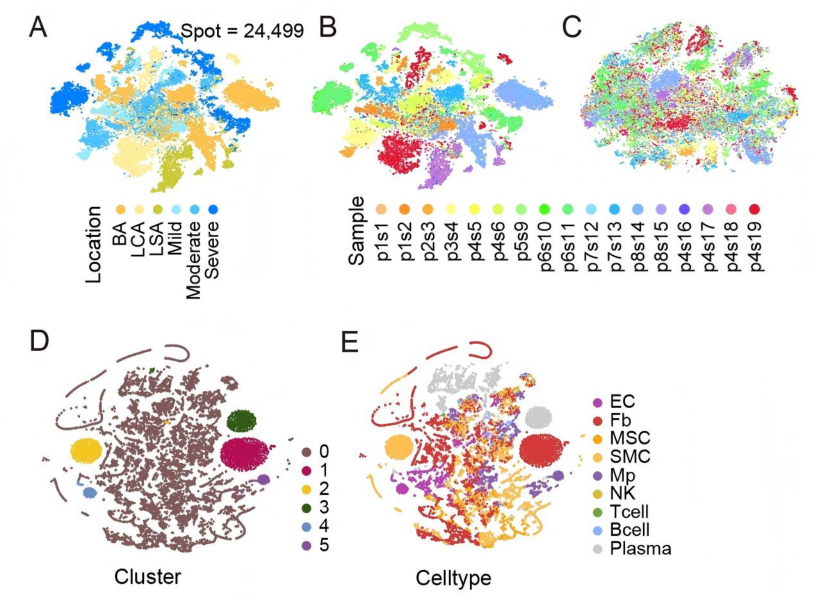

Figure S1

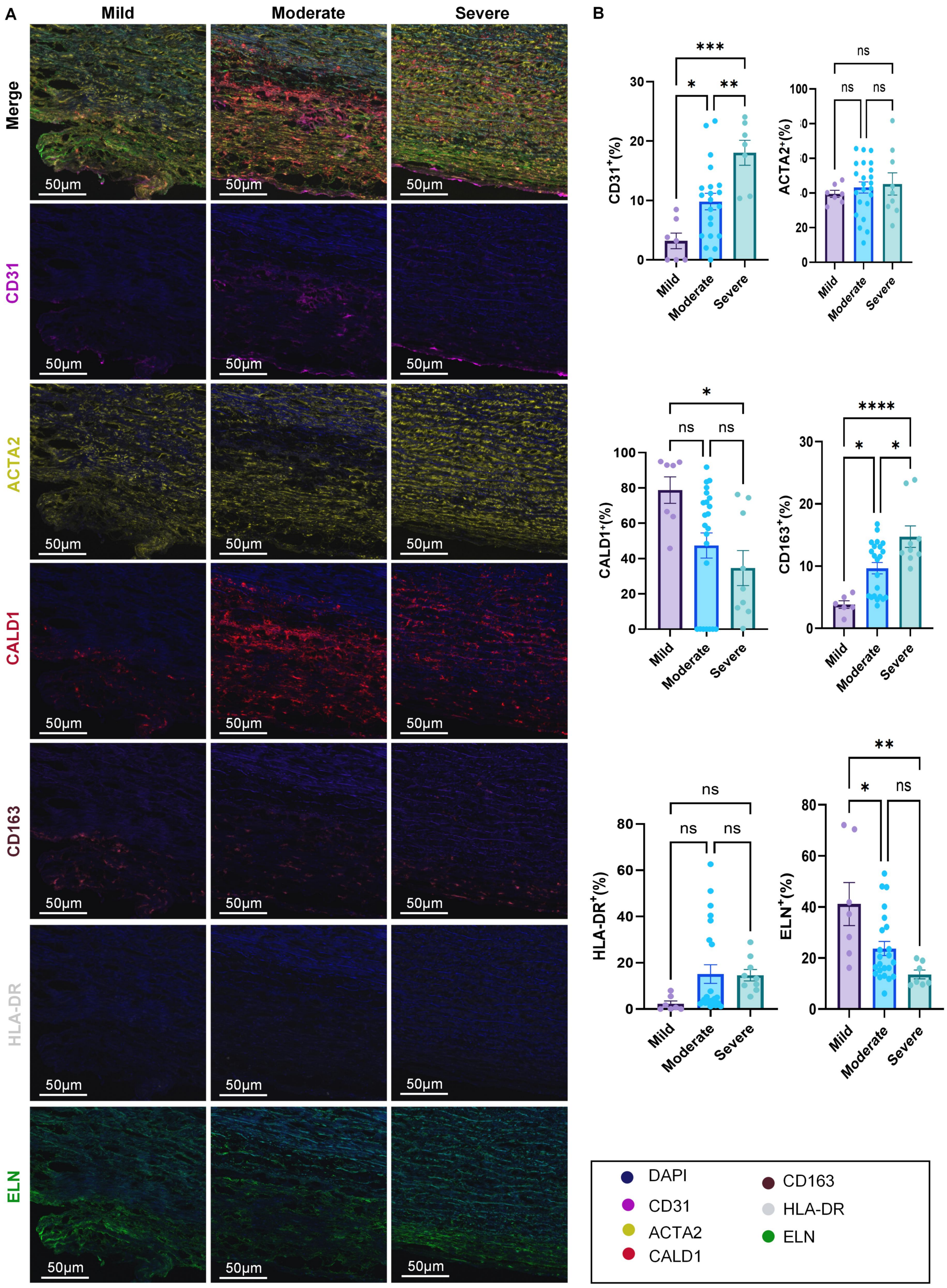

A
Mild
Moderate
Severe
Merge
CD31
ACTA2
CALD1
CD163
HLA-DR
ELN
50μm
B
CD31⁺(%)
ACTA2⁺(%)
CALD1⁺(%)
CD163⁺(%)
HLA-DR⁺(%)
ELN⁺(%)
ns
DAPI
CD31
ACTA2
CALD1
CD163
HLA-DR
ELN

Figure S2

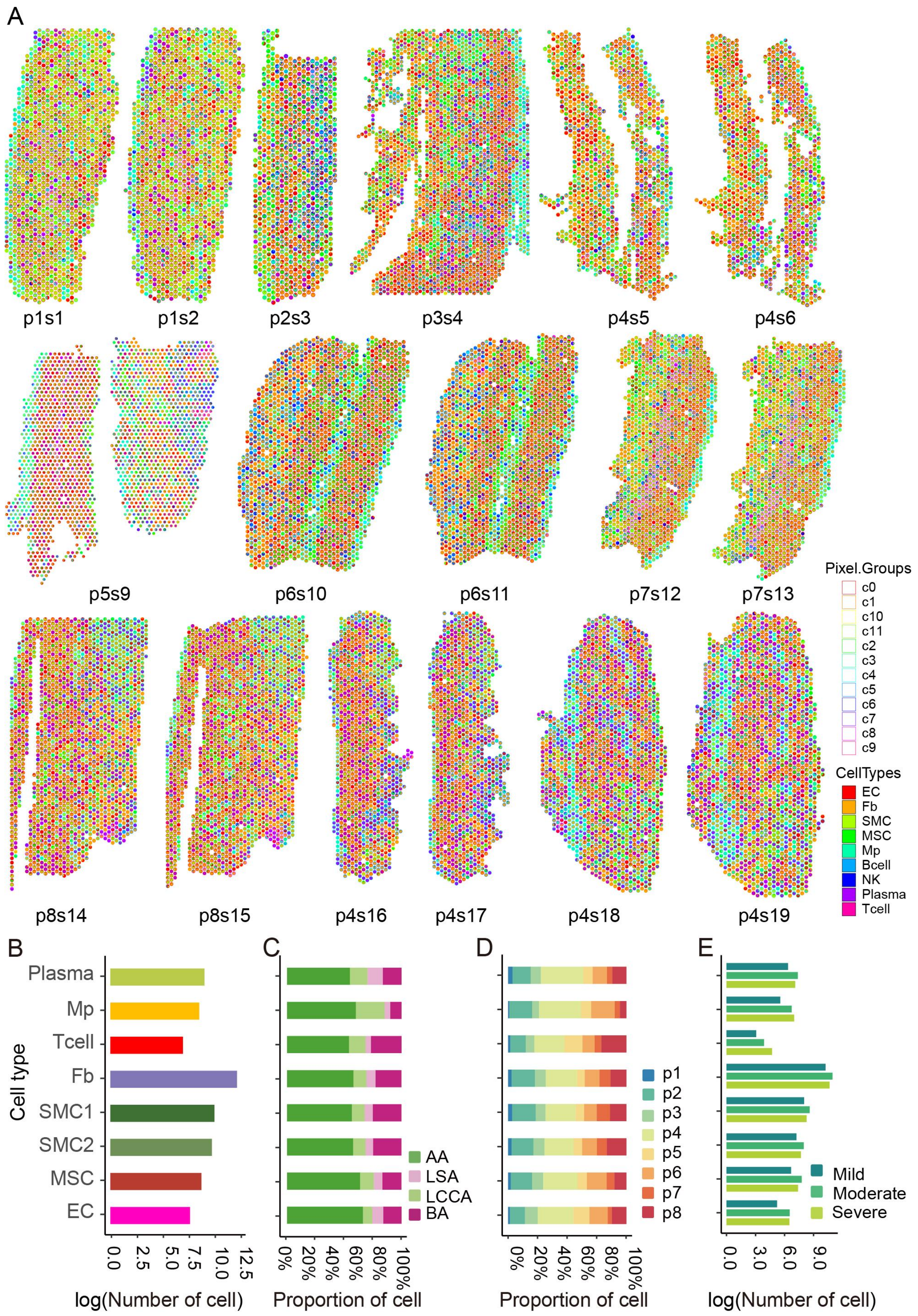
A
p1s1
p1s2
p2s3
p3s4
p4s5
p4s6
p5s9
p6s10
p6s11
p7s12
p7s13
p8s14
p8s15
p4s16
p4s17
p4s18
p4s19
Pixel.Groups
c0
c1
c10
c11
c2
c3
c4
c5
c6
c7
c8
c9
CellTypes
EC
Fb
SMC
MSC
Mp
Bcell
NK
Plasma
Tcell
B
Cell type
Plasma
Mp
Tcell
Fb
SMC1
SMC2
MSC
EC
0.0
2.5
5.0
7.5
10.0
12.5
log(Number of cell)
C
AA
LSA
LCCA
BA
0%
20%
40%
60%
80%
100%
Proportion of cell
D
p1
p2
p3
p4
p5
p6
p7
p8
0%
20%
40%
60%
80%
100%
Proportion of cell
E
Mild
Moderate
Severe
0.0
3.0
6.0
9.0
log(Number of cell)

Figure S3

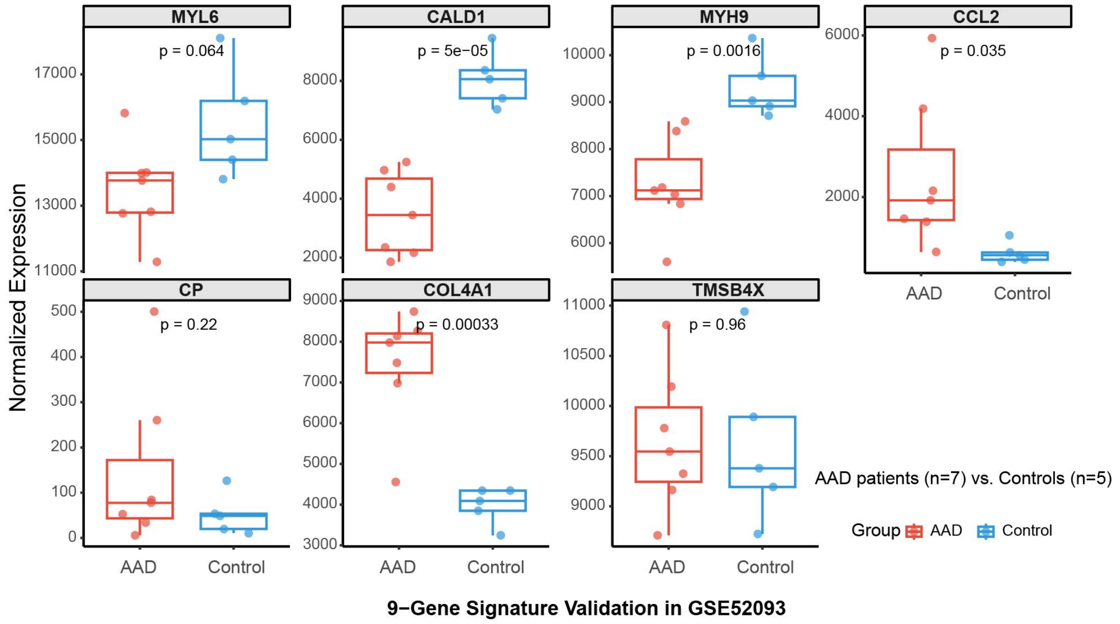

Figure S4

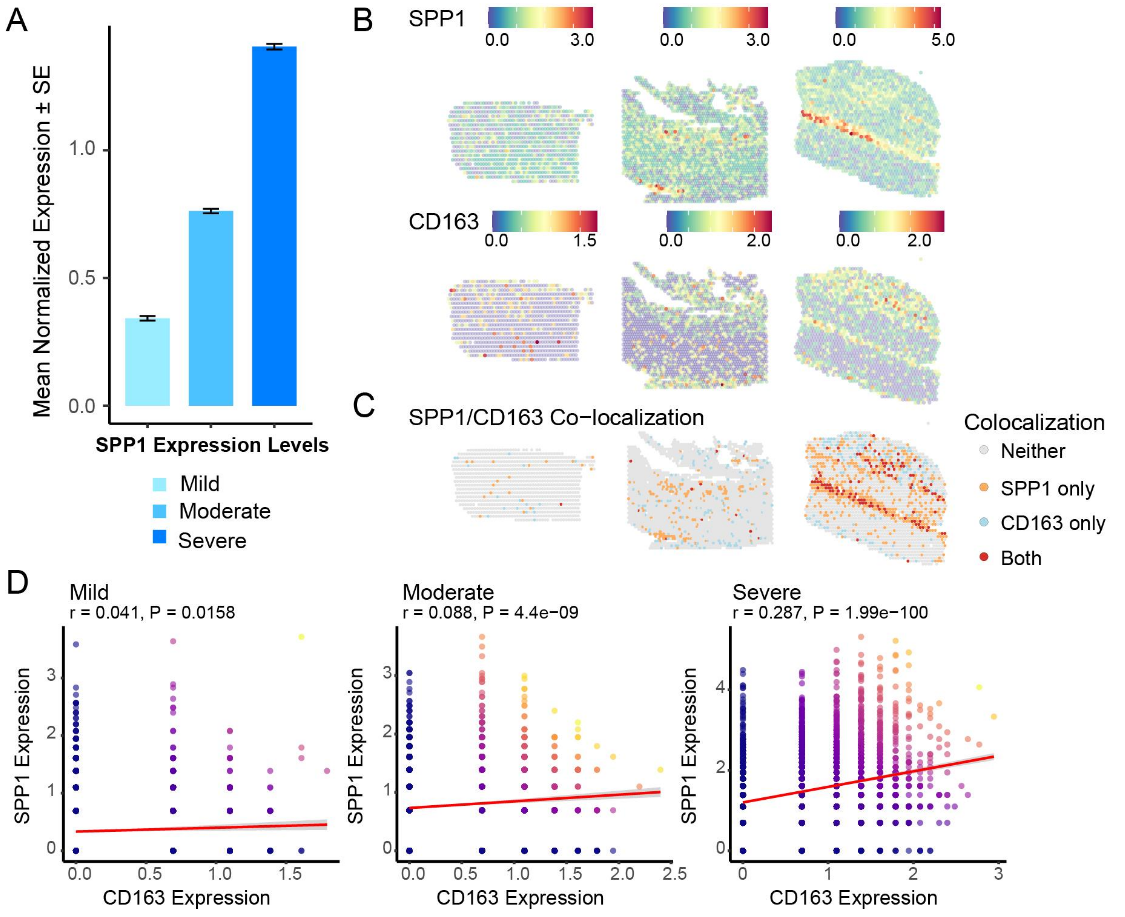

Figure S5

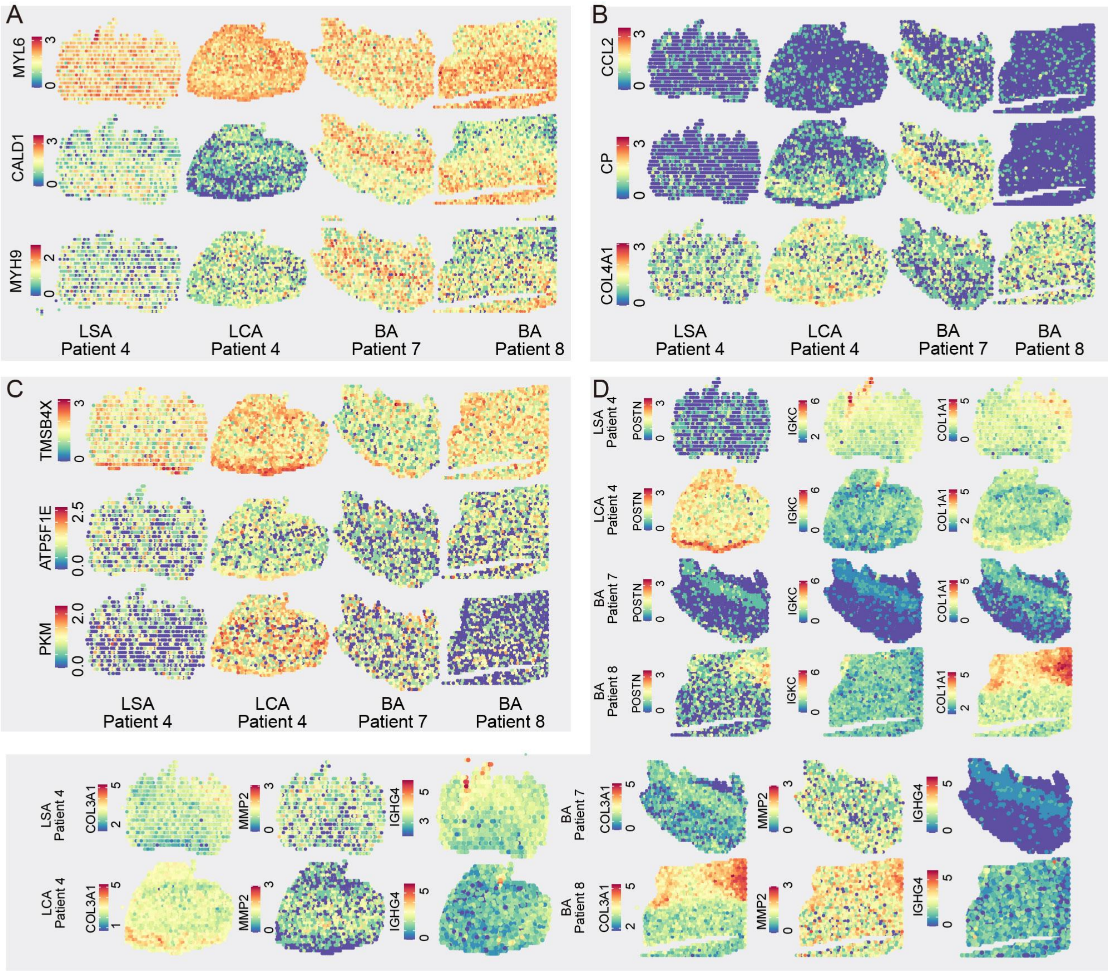
A
MYL6
CALD1
MYH9
LSA
Patient 4
LCA
Patient 4
BA
Patient 7
BA
Patient 8
B
CCL2
CP
COL4A1
LSA
Patient 4
LCA
Patient 4
BA
Patient 7
BA
Patient 8
C
TMSB4X
ATP5F1E
PKM
LSA
Patient 4
LCA
Patient 4
BA
Patient 7
BA
Patient 8
D
LSA Patient 4
LCA Patient 4
BA Patient 7
BA Patient 8
POSTN
IGKC
COL1A1
LSA Patient 4
LCA Patient 4
BA Patient 7
BA Patient 8
COL3A1
MMP2
IGHG4

Figure S6
